# Decoding epithelial-immune cell crosstalk in uropathogenic *Escherichia coli* urinary tract infections

**DOI:** 10.3389/fmicb.2026.1792758

**Published:** 2026-03-19

**Authors:** Zhenyu Jin, Zhenyu Zhou, Wenang Chen, Yan Xue, Zhengguo Zhang

**Affiliations:** 1 Department of Urology, The First Affiliated Hospital of Zhengzhou University, Zhengzhou, China; 2 Medical Research Center, The First Affiliated Hospital of Zhengzhou University, Zhengzhou, China

**Keywords:** epithelial cells, immune cells, innate immunity, urinary tract infections, uropathogenic *Escherichia coli*

## Abstract

Urinary tract infections (UTIs) pose a significant global health burden, characterized by a spectrum of clinical outcomes shaped by intricate epithelial-immune interactions. Uropathogenic *Escherichia coli* (UPEC) is the predominant etiological agent, accounting for the majority of cystitis cases. Upon breaching urothelial barriers, UPEC triggers a robust innate immune response involving cytokine secretion, antimicrobial peptide production, and recruitment of neutrophils and other immune cells. This review synthesizes current understanding of the dynamic crosstalk between urothelial and immune cells—highlighting mechanisms of bacterial recognition, phagocytosis, inflammatory mediation, and tissue repair—while addressing the limited adaptive immunity, which underlies the high recurrence rates. Elucidating these interactions provides a framework for developing targeted immunomodulatory therapies against UTIs.

## 1 Introduction

Urinary tract infections (UTIs) constitute a significant global health challenge, characterized by high morbidity and substantial economic burdens on healthcare systems (Zi et al., 2024; Gottlieb et al., 2025). Uropathogenic *Escherichia coli* (UPEC) serves as the predominant etiological agent, responsible for approximately 70–80% of infections across all clinical demographics. *Klebsiella pneumoniae, Staphylococcus saprophyticus, Enterococcus faecalis, group B Streptococcus (GBS), Proteus mirabilis* can also cause UTIs (Ronald, 2002; Flokas et al., 2016; Li et al., 2022). UTIs are a significant cause of morbidity in infant boys, older men and females of all ages (de Souza et al., 2023; He et al., 2025).

While UTIs encompass both lower (cystitis) and upper (pyelonephritis) tract infections, these distinct anatomical sites demand unique therapeutic and immunological considerations. Pyelonephritis carries the risk of life-threatening systemic complications; however r, cystitis accounts for the vast majority of cases (Ambite et al., 2021; Mancuso et al., 2023). Despite this high prevalence, our understanding of the bladder-specific immunological landscape remains limited. Building on this context, the present review focuses specifically on the innate and adaptive immune responses within the bladder during UPEC infection.

The bladder is a complex mucosal site that, despite being a non-lymphoid organ, exhibits remarkable heterogeneity in immune cell composition and function (Yu et al., 2019). Urothelial cells and immune cells in the bladder are critical for pathogen defense, maintaining epithelial barrier integrity, and preventing microbial dissemination. Their bidirectional crosstalk enables the bladder to maintain a delicate balance between rapid-response antibacterial immune reactions and resolving inflammatory cascades to maintain tissue homeostasis. This pattern explains why dysregulated urothelial immune communication can lead to chronic cystitis despite intact pathogen clearance mechanisms.

## 2 Orchestration of UTI defense by the bladder urothelium

The barrier function of the urothelium is critically important for the organism, as it prevents the infiltration of pathogens and harmful substances in urine, such as urea and other solutes (Dalghi et al., 2020). The urothelium is composed of three cell types: basal cells, intermediate cells, and superficial umbrella cells. Crucially, umbrella cells enforce barrier impermeability through high-resistance tight junctions (Whiteside et al., 2015). This barrier is further reinforced by specialized hexagonal uroplakin plaques that densely coat the apical membrane of umbrella cells, forming a crystalline protein matrix resistant to mechanical stress and pathogen adhesion (Jafari and Rohn, 2022). Complementing this physical barrier, a luminal glycosaminoglycan (GAG) layer mimics host cell receptors to competitively block UPEC adhesion (Hurst, 1994). Functionally, these structural defenses operate in concert with the hydrodynamic force of urinary flow, which serves to mechanically flush unbound bacteria from the bladder lumen.

**Figure 1:**
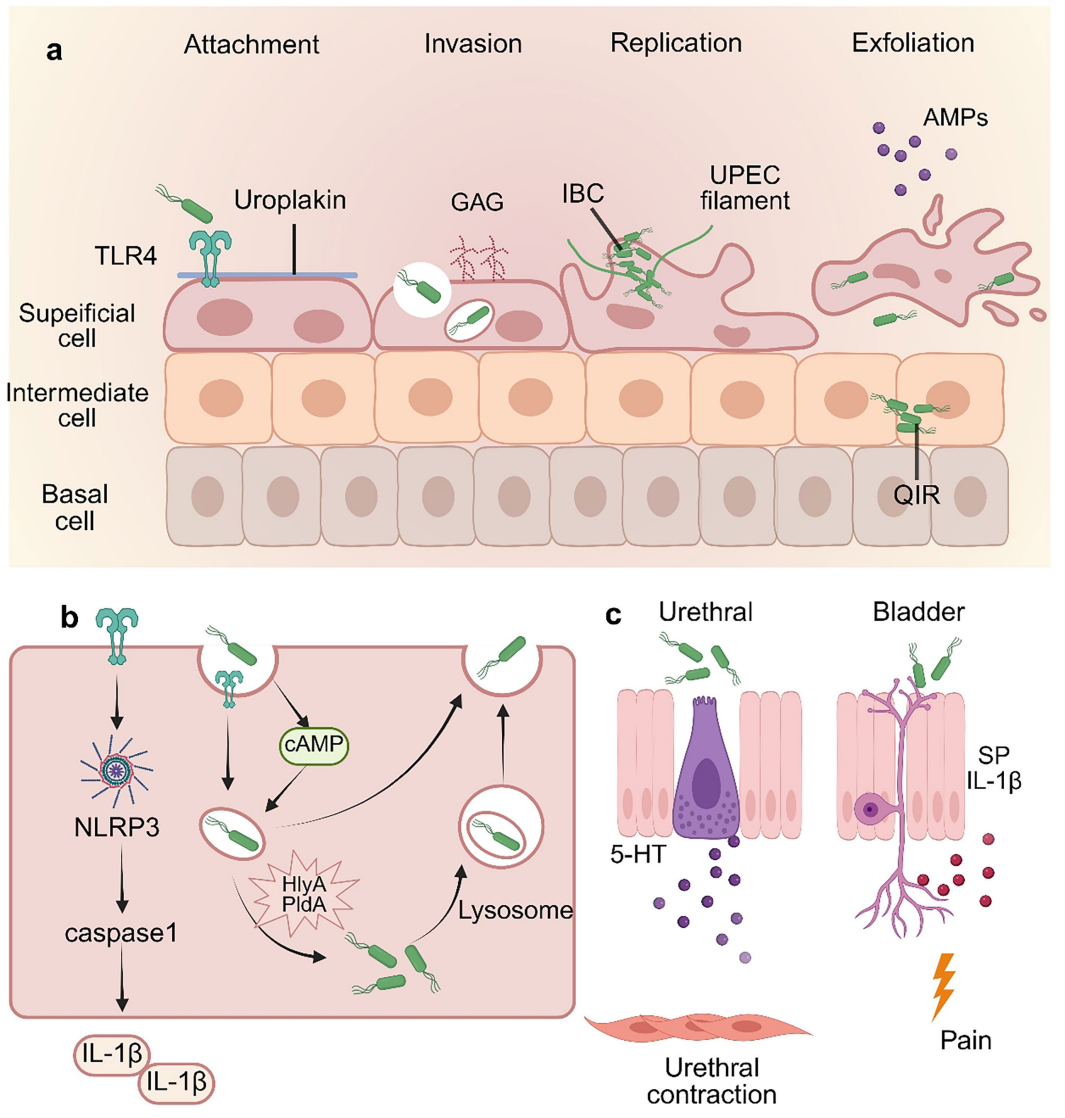
Interaction between host innate defense and UPEC in the bladder. **(a)** The urothelium consists of three cell types: basal, intermediate, and superficial umbrella cells. Under homeostatic conditions, the mucosa defends against invasion through a multilayered system, including apical uroplakin plaques, a glycosaminoglycan (GAG) layer, and secreted antimicrobial peptides. To breach this defense, UPEC utilizes adhesive virulence factors to invade epithelial cells. Following internalization, UPEC can proliferate to form intracellular bacterial communities (IBCs). However, adhesion or invasion may trigger apoptotic pathways in epithelial cells, leading to exfoliation and clearance of infected cells. To escape immune clearance, UPEC can differentiate into quiescent intracellular reservoirs (QIRs), enabling long-term persistence within the bladder mucosa. Conversely, the host attempts to eliminate infected cells via regulated apoptosis and exfoliation. **(b)** Bladder epithelial cells mediate immune responses via Toll-like receptors (TLRs). They can activate caspase-1-mediated inflammatory cell death to rapidly eliminate intracellular bacteria. TLR activation also increases intracellular cAMP levels. This triggers exocytosis of RAB27b^+^ vesicles, expelling intracellular UPEC back into the bladder lumen. Some UPEC can escape into the cytoplasm. These cytosolic bacteria are rapidly captured by autophagosomes and delivered to lysosomes, forming autolysosomes. The majority of these bacteria-containing compartments are then expelled from the cell via lysosomal exocytosis. However, UPEC employs virulence factors, such as HlyA and PldA to disrupt these processes and facilitate long-term intracellular survival. **(c)** UTIs trigger complex neural responses within the urinary tract. Bladder epithelial cells and neuronal cells release signaling molecules that amplify bladder pain responses, thereby prompting the body to react to UPEC infection. Within the urethral epithelium, neuroendocrine cells secrete serotonin (5-HT), which stimulates the contraction of urethral smooth muscle, thereby facilitating the active expulsion of bacteria.

The molecular mechanisms underlying UPEC invasion and persistence have been extensively characterized (Figure 1a). The process of UPEC entering bladder epithelial cells begins with the binding of the FimH adhesin on type 1 pili to mannosylated uroplakins and α3β1 integrin on umbrella cells (Sauer et al., 2016). Upon binding to α3β1 integrin, FimH triggers Rho-family GTPases, leading to local actin rearrangement and bacterial internalization (Martinez and Hultgren, 2002). While initial FimH binding is low-affinity, urinary shear forces induce a “catch-bond” conformational change that dramatically strengthens this interaction, preventing bacterial washout (Dumenil et al., 2020). Beyond the pilus system, UPEC utilizes virulence strategies such as metal ion scavenging systems and biofilm formation to establish infections while producing toxins including hemolysins A (HlyA) and cytotoxic factors that disrupt epithelial barriers, enabling tissue invasion (Zhang et al., 2021; Pakbin et al., 2025; Nagamatsu et al., 2015). During intracellular invasion, UPEC can internalize, proliferate within host cells, form intracellular bacterial communities (IBCs), and protrude into the bladder lumen (Anderson et al., 2003). IBCs protect bacteria from antibiotics and host immune responses, aiding persistence in UTIs. The cycle repeats when infected cells rupture, releasing filamentous UPEC into the urine to infect neighboring cells. This filamentous morphology enables UPEC to resist neutrophil-mediated killing and survive extracellularly (Coers et al., 2022). Alternatively, a subset of bacteria may invade the underlying urothelium to establish quiescent intracellular reservoirs (QIRs) (Sharma et al., 2021; Mysorekar and Hultgren, 2006), which remain viable for extended periods and evade antibiotic treatment. The sequential progression and molecular pathogenesis of kidney infections remain poorly understood, primarily due to the lack of robust animal models for severe pyelonephritis, hindering *in vivo* investigations.

Upon breaching constitutive passive defenses, UPEC is immediately detected by the bladder epithelium, initiating a multi-pronged antimicrobial response. Bladder epithelial cells detect bacteria via pattern recognition receptors (PRRs), triggering inflammatory responses against UPEC. Among PRRs, Toll-like receptors (TLRs) are the most extensively studied in UTIs (Song and Abraham, 2008) (Figure 1b). These receptors recognize pathogen-associated molecular patterns (PAMPs) and damage-associated molecular patterns (DAMPs), inducing transcription of proinflammatory cytokines/chemokines and upregulating costimulatory molecules on antigen-presenting cells (Tan et al., 2023). TLR4, which detects UPEC-derived lipopolysaccharide (LPS), is widely regarded as a key mediator in host defense against UTIs (Song et al., 2007). A variety of animal studies demonstrate that mice defective in LPS signaling exhibit markedly reduced neutrophil infiltration, resulting in higher bacterial loads than controls (Edén et al., 1988; Hagberg et al., 1984). Additional studies link reduced TLR4 expression to asymptomatic bacteriuria (Ambite et al., 2021). TLR4 activates multiple signaling pathways in bladder epithelial cells, including NF-κB activation that promotes the release of cytokines (e.g., IL-6, IL-8) and inflammatory mediators (Ching et al., 2018). TLR4 also associates with intracellular calcium influx, rapidly elevating cAMP levels via adenylate cyclase 3 (AC3)—a process facilitating bacterial expulsion (Song et al., 2007; Bishop et al., 2007). This latter pathway is critical for coordinating the mechanical expulsion of bacteria described below.

Urothelial cells and recruited leukocytes produce multiple antimicrobial peptides (AMPs), which represent a key component of the host innate immune response, exhibiting both bactericidal and immunomodulatory functions. Uromodulin (Tamm-Horsfall protein), produced by the kidneys, is the most abundant protein in urine (Pak et al., 2001). Upon binding to UPEC, it prevents bacterial-epithelial interactions by inducing bacterial aggregation and adhesion blockade (Pak et al., 2001). Other key AMPs include pentraxin 3 (PTX3), RNase7, Nur77and secretory leukocyte protease inhibitor (SLPI). PTX3 binds to bacterial surfaces to promote complement-mediated killing and enhance macrophage phagocytosis (Jaillon et al., 2014). RNase7 exerts dual antimicrobial and anti-inflammatory effects by directly lysing bacteria while inhibiting JAK/STAT signaling and TLR4-dependent IL-6 and IL-8 production (Ho et al., 2022). Nur77 is a nuclear receptor that acts as an intracellular sensor for bacterial LPS. Upon activation, it restricts UPEC invasion by inhibiting endocytosis and promotes the expulsion of intracellular bacteria from urothelial cells, representing a novel antibiotic-sparing target for UTIs treatment (Collins et al., 2024). SLPI is a multifunctional AMP with antiprotease, antimicrobial, and immunomodulatory properties that plays a protective role in urinary tract defense. Recent studies have shown that UPEC-infected SLPI-deficient mice exhibit higher UPEC burden, prolonged bladder inflammation, and elevated urinary neutrophil elastase (NE) levels during infection, confirming its critical role in bacterial control and resolution of inflammation (Rosen et al., 2024).

During bladder filling or emptying, urothelial cells dynamically modulate surface area through cAMP-dependent endocytosis and exocytosis of subapical discoidal/fusiform vesicles (DFVs), enabling morphological adaptation to changing bladder volume (Wu et al., 2025). Following UPEC invasion, bacteria are entrapped within RAB27b^+^ fusiform vesicles (Chen et al., 2003). There are two distinct expulsion mechanisms in response to infection: First, at 4–6 h post-infection, TLR4 signaling elevates intracellular cAMP to trigger exocytosis of Rab27b^+^ vesicles to eliminate intracellular bacteria (Bishop et al., 2007). Second, cytosolic UPEC is captured by autophagosomes for lysosomal delivery; after autophagosome-lysosome fusion, bacteria are expelled from lysosomes via transient receptor potential cation channel subfamily M member 3 (TRPML3)-dependent exocytosis (Miao et al., 2015). Unlike the first pathway, this membrane-encased expulsion prevents UPEC reattachment to the bladder epithelium and ensures bacterial elimination from urinary tract. To subvert these expulsion mechanisms, UPEC can utilize virulence factors such as HlyA to escape from RAB27b^+^ vesicles, disrupt host microtubules, and inhibit lysosomal acidification (Naskar et al., 2023). Concurrently, outer membrane phospholipase PldA degrades vesicular phospholipids to facilitate cytosolic escape and reduces host phosphatidylinositol 3-phosphate (PI3P) levels to stall pre-autophagosomal maturation, thereby evading autophagic capture and subsequent lysosomal exocytosis (Li et al., 2024a; Pang et al., 2022).

As a final defense mechanism when other strategies fail, the bladder employs epithelial exfoliation to reduce bacterial burden. FimH^+^ UPEC triggers a rapid caspase-dependent apoptosis-like pathway in host cells, leading to DNA fragmentation and exfoliation of infected superficial urothelial cells (Mulvey et al., 1998). Additionally, bladder epithelial cells secrete IL-1β to recruit mast cells. While endocytosing mast cell granules, bladder epithelial cells undergo lytic death (Choi et al., 2016). This process is precisely regulated by inflammasome-associated proteins. A study has revealed that caspase-1, caspase-4, and NLRP3 differentially orchestrate the bladder epithelial cells response to UPEC infection. They control not only the IL-1β release and pyroptosis but also the expression of various other inflammatory mediators, thereby distinctly influencing downstream immune effector functions such as neutrophil phagocytosis and reactive oxygen species (ROS) production (Lindblad et al., 2022). Following exfoliation of infected cells, the underlying tissues are then subjected to toxic urinary components. To mitigate these effects, epithelial loss rapidly triggers quiescent urothelium to enter a hyperproliferative state, restoring the epithelial barrier (Shin et al., 2011). Concurrently, UPEC employs multiple strategies to suppress urothelial exfoliation, primarily to facilitate intracellular replication and evade host innate immunity. For instance, UPEC secretes Nucleoside-diphosphate kinase (NDK) to inhibit caspase-1-dependent pyroptosis by consuming extracellular ATP, thereby preventing urothelial exfoliation. The ndk expression is activated in response to intracellular ROS, which is sensed by UPEC via ROS sensor OxyR (Li et al., 2024b).

UTIs-induced bladder irritative symptoms—frequency, urgency, and dysuria—cause significant patient distress. Urothelial cells exhibit neuron-like properties, possessing sensory and signaling capabilities that enable responses to mechanical and chemical stimuli. These cells secrete multiple signaling molecules, including neurotrophins, neuropeptides, prostaglandins, and cytokines (Jafari and Rohn, 2022). During UTIs, these mediators communicate with bladder neurons, smooth muscle cells, and inflammatory cells to combat infection. Substance P (SP) and its receptor Neurokinin-1 (NK1R) mediate nociception and pain signaling (Chien et al., 2003). UPEC stimulates SP/NK1R expression in both urothelial cells and neurons, which concurrently secrete IL-1β to amplify inflammation and pain responses (Butler et al., 2018). Recent research demonstrates that urethral neuroendocrine cells produce serotonin (5-HT) upon LPS detection, inducing urethral smooth muscle contraction and bacterial expulsion. Defects in this pathway may compromise urethral barrier defense and contractility, thereby leading to bacterial colonization of the bladder and increased susceptibility to UTIs (Ambrogi et al., 2025) (Figure 1c).

Collectively, recent studies point to novel pathways for understanding the molecular mechanisms underlying urothelial behavior during and after UTIs, offering potential avenues for therapeutic modulation. Despite extensive research on the urothelium, key knowledge gaps persist. Active areas of investigation include the heightened bacterial susceptibility in older adults, disparities in UTI incidence and recurrence rates between sexes, and the impact of sex hormones on infection dynamics.

## 3 The function of immune cells during UTIs

### 3.1 Innate immune cells

**Figure 2:**
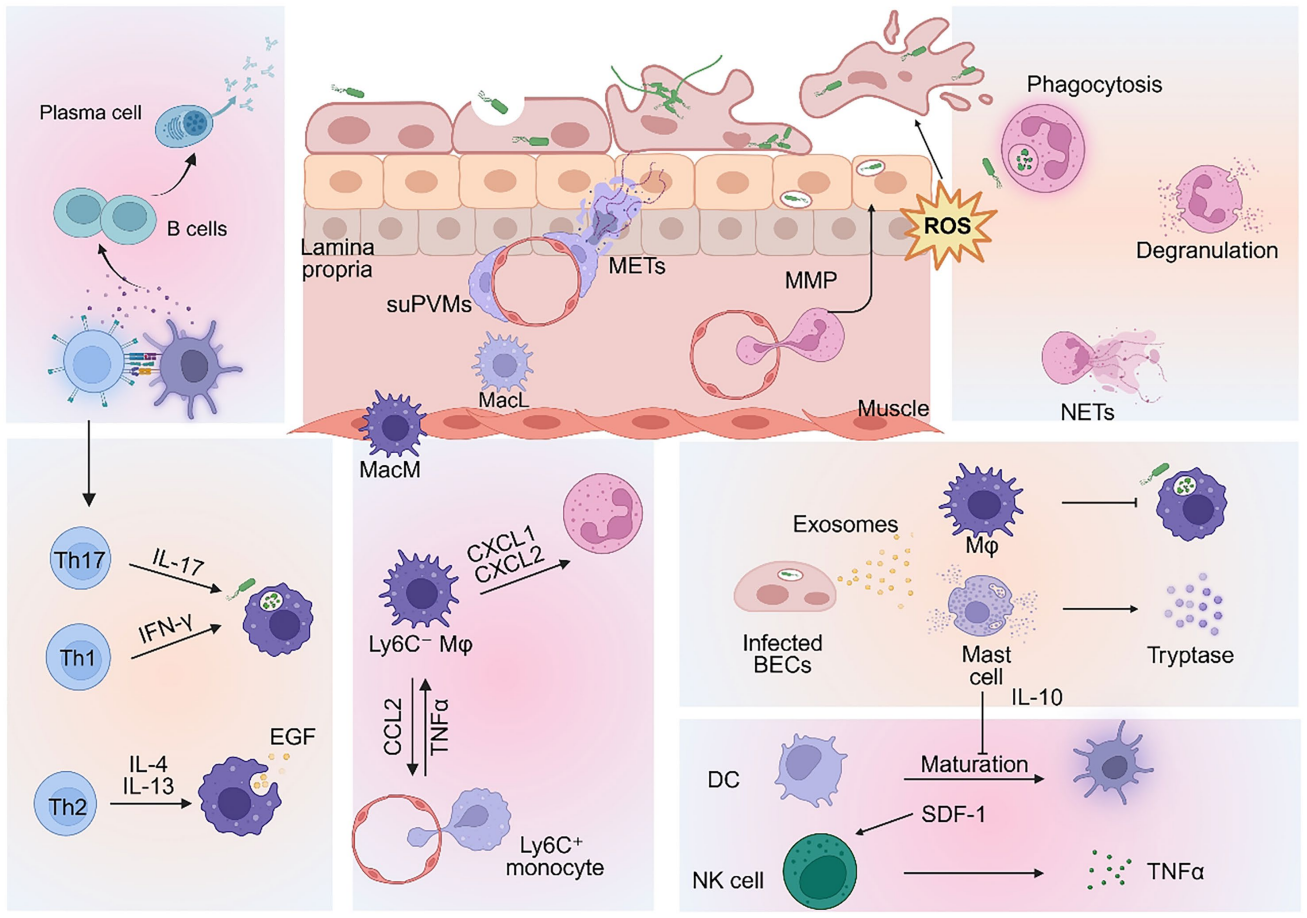
Epithelial-immune coordination of antibacterial defense in the bladder. Bladder epithelial cells release various cytokines to coordinate immune cells recruitment and antibacterial responses. Neutrophils clear bacteria through phagocytosis, degranulation, and neutrophil extracellular traps (NETs). However, neutrophil transepithelial migration and phagocytosis require reactive oxygen species (ROS), which contribute to epithelial exfoliation. During infection, macrophage subsets exhibit functional heterogeneity through coordinated crosstalk. Resident Ly6C^−^ macrophages secrete CXCL1 and CCL2 to recruit neutrophils and Ly6C^+^ monocytes. Subsequently, TNF-α signaling from these recruited monocytes stimulates resident macrophages to produce CXCL2, thereby promoting neutrophil transepithelial migration. Tissue-resident MacM and MacL macrophages respond differently to UPEC, participating in antigen presentation and bacterial phagocytosis. Suburothelial perivascular macrophages (suPVMs) form a vascular-immune barrier and release macrophage extracellular traps (METs) to limit pathogen spread. UPEC-infected epithelial cells release exosomes that exacerbate infection by inducing macrophage apoptosis and suppressing phagocytosis. Epithelial-derived exosomes also stimulate mast cells to produce tryptase, promoting epithelial shedding. Mast cell-derived IL-10 inhibits the maturation of dendritic cells (DCs), thereby suppressing subsequent lymph node-mediated immune responses. NK cells are recruited via SDF-1 and exert bactericidal activity through perforin secretion. T cells differentiate into multiple subsets, including Th1, Th2, and Th17 cells. Th1 and Th17 cells enhance macrophage phagocytosis, while Th2 cells promote tissue repair. B cells proliferate in the submucosa and mature into IgA-producing plasma cells.

The innate immune system in the bladder consists of resident cells and recruited immune components. During infection, resident cells rapidly produce inflammatory cytokines to drive a strong proinflammatory response while recruiting effector immune cells from the blood. This coordinated process highlights the dynamic complexity of host defense mechanisms against UTIs. In this section, we provide an overview of the major innate immune cells of the urinary tract, detail their specialized antimicrobial functions, and highlight the key communication networks between the immune cells themselves and with the bladder epithelium that coordinate protective responses (Figure 2).

#### 3.1.1 Neutrophils

Neutrophils are the primary and first-recruited innate immune cells in UTIs, clearing pathogens via bactericidal mechanisms. Their recruitment is initiated by inflammatory mediators from tissue-resident leukocytes or cytokines secreted from epithelial cells via activation of TLR4 signaling (Haraoka et al., 1999). Neutrophil infiltration typically peaks 6 h post-infection and is predominantly driven by pro-inflammatory cytokines such as CXCL1, CXCL8 (IL-8), and IL-1 (Agace et al., 1993; Godaly et al., 2000; Duell et al., 2012). Moreover, granulocyte colony-stimulating factor (G-CSF), secreted during infection, promotes neutrophil influx into the bladder, while neutralizing G-CSF reduces this migration. Paradoxically, G-CSF depletion also decreases bacterial burden which may be mediated by enhancing macrophage activating cytokine secretion, ultimately shifting the bladder immune response toward a more favorable outcome for the host (Ingersoll et al., 2008).

Besides phagocytizing bacteria at infection sites, neutrophils also deploy additional antimicrobial strategies: releasing granule proteins (elastase, antimicrobial peptides) and extruding neutrophil extracellular traps (NETs) – chromatin structures coated with bactericidal proteins that entrap pathogens while minimizing host tissue damage (Yu et al., 2017; Kraus and Gruber, 2021; Krivosikova et al., 2023). Experimental models have shown that *PAD4^−^/^−^* mice, which have reduced NETs formation, have significantly increased bacterial loads *in vivo* (20-fold in the bladder and 300-fold in the kidneys), highlighting the protective role of NETs (Krivosikova et al., 2023). As noted above, UPEC counteracts these defenses through filamentation, biofilm-like capsule formation, and virulence factors such as TcpC, which inhibits NETs formation and suppresses neutrophil recruitment (Ou et al., 2021).

The ROS plays a critical role in neutrophil-mediated bacterial clearance. However, excessive ROS production is detrimental to the host, as elevated levels correlate with worsening bladder infection (Lindblad et al., 2022). ROS are also implicated in neutrophil transepithelial migration across the bladder epithelium and are essential for the subsequent mucosal damage. After leaving the blood vessels, recruited neutrophils need to cross the lamina propria and basement membrane of bladder epithelial cells (Zec et al., 2016). During tissue migration, activated neutrophils secrete matrix metalloproteinase 9 (MMP-9) to penetrate the basement membrane to enter the superficial epithelium and bladder lumen. However, this process—combined with the generation of ROS—entails the risk of urothelial damage and pathological exfoliation, which may facilitate recurrent infections. Emerging evidence suggests that macrophage extracellular traps (METs)-associated MMP13 is also involved in neutrophil migration, although the coordination between MMP13 and MMP9 remains unclear (Li et al., 2025).

Host-secreted AMPs synergize with neutrophil function. The absence of PTX3 impairs neutrophil phagocytosis and phagosome maturation, consequently leading to an increased bacterial burden in the bladders and kidneys of (*Ptx3^−^/^−^*) mice (Jaillon et al., 2014). Tamm-Horsfall protein (THP), via its terminal sialic acid, binds the inhibitory receptor Siglec-9 (Patras et al., 2017). This interaction suppresses neutrophil activities, including chemotaxis and ROS release, thereby preventing inflammatory damage. Furthermore, during infection, THP can coordinate with NETs to achieve potent antimicrobial effects, preventing bacterial ascension to the kidneys (Mercado-Evans et al., 2025). *In vitro* lactoferrin does not directly inhibit UPEC growth but reduces bacterial adhesion to epithelial cells and enhances neutrophil bactericidal capacity and NETs production (Patras et al., 2019). Intravesical injection of exogenous lactoferrin significantly reduces bacterial burden and neutrophil infiltration in mouse bladders. Lipocalin-2 (LCN2, also known as NGAL) is a specific antibacterial protein produced by epithelial cells and neutrophils in response to TLR4-mediated bacterial sensing during bladder and kidney infections in mice (Chen et al., 2022). LCN2 binds and inactivates enterobactin, thereby neutralizing bacterial siderophore-mediated iron acquisition. Beyond this iron-sequestering role, LCN2 has also been shown to suppress UPEC infection and attenuates inflammatory responses under hyperglycemic conditions via modulation of the JAK/STAT signaling pathway (Chen et al., 2022). Clinically, LCN2 levels in urine and plasma of patients with acute UTIs can be used as a sensitive diagnostic tool, allowing physicians to quickly identify the severity of infection (ElNahal et al., 2025).

#### 3.1.2 Macrophages

As the most abundant innate immune cells in the bladder, macrophages play critical roles in bacterial clearance and inflammation regulation during UPEC infection. Historically, macrophages were often simplistically categorized as pro-inflammatory (M1) or anti-inflammatory (M2) (Zhang et al., 2020; Wang et al., 2014; Wang et al., 2025). However, with advancing understanding of macrophage heterogeneity, immunologists now recognize this model as too limited (Nahrendorf and Swirski, 2016). During UPEC infection, the bladder macrophage pool comprises two main subsets: tissue-resident macrophages and monocyte-derived macrophages (which infiltrate tissues in response to inflammatory signals like cytokines) (Lacerda Mariano and Ingersoll, 2018). Under steady-state conditions, tissue-resident macrophages primarily self-renew through local proliferation. During inflammation, they are often replaced by monocyte-derived counterparts. Tissue-resident macrophages serve as the primary antigen-presenting cells during UTIs. They phagocytose bacteria and engage in crosstalk with epithelial and other immune cells via cytokine and chemokine production (Lacerda Mariano and Ingersoll, 2018).

Furthermore, tissue-resident macrophages are not homogeneous but exhibit phenotypic and functional plasticity, adapting their precise profile based on diverse factors within the local microenvironment (Jasmine et al., 2025; Liu et al., 2025). A study reveals functional specialization of bladder resident macrophages utilizing single cell RNA sequencing: muscularis macrophages (MacM) demonstrate heightened phagocytic activity, while lamina propria macrophages (MacL) display a distinct transcriptome marked by upregulated TLR signaling and chemokine production, suggesting greater inflammatory potential (Mariano et al., 2020). Furthermore, a recent study has delineated that a subset of MacL tightly adheres to bladder laminal propria capillaries as suburothelial perivascular macrophages (suPVMs), which prevent bacteremia and systemic dissemination of UPEC during acute cystitis. Through crosstalk with inflamed endothelium by Mac-1/ICAM-1 signaling, activated suPVMs subsequently undergo METosis, extruding macrophage extracellular traps (METs) into the urothelial compartment, thereby physically sequestering uropathogens and preventing trans-urothelial dissemination (Li et al., 2025). Moreover, in a UPEC elicited cystitis mouse model, a subset of macrophages, CD137L^+^ macrophages, identified by single-cell RNA sequencing has been demonstrated to play a crucial role in attenuating excessive inflammatory response by regulating Tregs (Liu et al., 2025). In addition, our group also identified a novel subset of testicular macrophage as S100a4^+^ macrophages, which transitioned to a myofibroblast-like phenotype, producing extracellular matrix proteins to drive fibrotic disease in UPEC-induced epididymo-orchitis (Wang et al., 2025). Although primarily characterized in epididymo-orchitis, similar fibrotic mechanisms may be relevant to chronic sequelae of UTIs. Therefore, advancing technologies are enabling the identification of an increasing number of macrophage subsets with distinct functional properties.

Mechanistically, macrophages initiate immune response by activation of TLRs signaling pathway when detecting invading pathogens. CD14 as a coreceptor of PRRs and orchestrates macrophage-mediated UPEC clearance by modulating the release of cytokines (e.g., CXCL2, IL-1β, and IL-10) (Carey et al., 2016; Wang et al., 2022). Inflammasomes within macrophages further amplify immunity—different UPEC strains variably activate inflammasomes in human and murine macrophages, triggering IL-1β release and pyroptosis. However, UPEC can also counteract this process through its virulence factor TcpC, which binds NLRP3 and caspase-1 to suppress inflammasome activation and IL-1β production (Fang et al., 2022). Furthermore, the communication between the bladder epithelial cells and macrophages during UPEC infection may affect immune outcomes. Epithelial-derived IL-6 induces CX3CL1 production, recruiting macrophages to infected urothelium for bacterial phagocytosis (Bottek et al., 2020). Additionally, we have found the communication of UPEC and bladder epithelial cells via exosomes. UPEC infection induces bladder epithelial cells secretion of exosomes enriched with miR-18a-5p, which are subsequently taken up by macrophages. These exosomes further trigger TNF-*α* production via the PTEN/MAPK/JNK pathway, leading to enhanced inflammation, increased macrophage apoptosis, and impaired phagocytosis. Consequently, bacterial clearance is reduced, exacerbating UTI severity and tissue damage (Wang et al., 2024).

Other inflammatory mediators released by macrophages contribute to leukocyte recruitment for host defense during UTIs. Ly6C^−^ tissue-resident macrophages produce cytokines CXCL1 and CCL2 to recruit circulating neutrophils and Ly6C^+^ monocytes (Schiwon et al., 2014). These recruited monocytes subsequently generate TNF-α, which induces CXCL2 production by Ly6C^−^ macrophages, driving neutrophil infiltration and bacterial phagocytosis (Schiwon et al., 2014). However, other studies indicate that depleting resident macrophages does not alter cytokine expression post-UPEC infection and minimally affects bacterial loads (Mora-Bau et al., 2015; Hannan et al., 2014). This suggests that urothelial cells serve as a potent compensatory source of inflammatory mediators, ensuring that host defense remains robust even when macrophage signaling is compromised.

A major challenge in bladder macrophage research is distinguishing tissue-resident macrophages from monocyte-derived macrophages during infection. This difficulty arises because recruited monocytes upregulate surface markers typically from resident macrophages while downregulate monocyte-specific proteins, and these subsets differentially impact adaptive immunity. Another challenge is the limited yield of bladder macrophages from murine models, compelling researchers to use bone marrow-derived macrophages (BMDMs) or peritoneal macrophages instead. However, biological differences stemming from tissue-resident macrophage heterogeneity warrant significant attention. It is reasonable to postulate that these variations may drive distinct macrophage responses across various pathologies. Therefore, elucidating the origin, distribution, and functional heterogeneity of bladder-resident macrophages—even the impact of circadian rhythms—is crucial for future research (Wang et al., 2022).

#### 3.1.3 Mast cells

Mast cells are tissue-resident granulocytes that inhabit most organs and play critical defensive and immunomodulatory roles at barrier sites. In the healthy human bladder, mast cells are distributed throughout the urothelium, lamina propria, and detrusor muscle layers, with particularly low numbers in the urothelium and lamina propria (Tauber et al., 2023). In murine models, however, mast cells are more prominent in the lamina propria and detrusor muscle (Smith et al., 2024). In both species, a subset of mast cells resides near blood and lymphatic vessels, a feature that may inform their different functional roles in bladder pathology. These cells store preformed proinflammatory mediators—including chymase, tryptase, TNF-α, histamine, CXCL1, and CXCL2—within cytoplasmic granules. Significant amounts of histamine can be detected in the urine as early as 30 min after bladder infection in mice (Abraham et al., 2001). During UPEC infection, mast cell degranulation triggers diverse physiological responses to mediate active immune defense. Mast cell-deficient mice exhibit delayed bacterial clearance due to impaired neutrophil recruitment and activation compared to wild-type counterparts (Abraham et al., 2001). Emerging evidence indicates that UPEC infection induces pyroptosis of bladder epithelial cells and results in releasing exosomes containing IL-1β and IL-18, consequently promoting mast cell migration. Mast cells further compromise bladder epithelial barrier integrity through the tryptase-PAR2 axis (Wu et al., 2019).

Mast cells exhibit unique dynamic regulatory properties during bladder infections. During early phase of infection, they directly promote pathogen clearance by inducing epithelial exfoliation (Choi et al., 2016). However, following infection progresses, these cells undergo a functional shift. During late phase of infection, activated mast cells release IL-10, which is a potent immunosuppressive cytokine that inhibits the activation of dendritic cells and the subsequent responses of T cells and B cells. Murine studies demonstrate that mast cell-specific IL-10 deletion reduces persistent infection, whereas wild-type controls maintain higher bacterial loads through IL-10-mediated suppression of adaptive immunity (Chan et al., 2013). Elevated urinary IL-10 levels are also observed in elderly patients with recurrent UTIs (Drage et al., 2019). This functional switch carries physiological rationale: early-stage mast cell-driven exfoliation eliminates pathogens but compromises bladder barrier function, causing urine-induced pain, while late-stage IL-10 release suppresses dendritic cells co-stimulatory molecule expression (impairing antigen presentation in draining lymph nodes) and promotes epithelial regeneration (Shelburne et al., 2009). This dual regulation prevents excessive adaptive responses to infection while restoring tissue homeostasis. However, it remains unclear whether this functional switch in mast cells arises from distinct developmental subpopulations or from intrinsic genetic reprogramming during infection.

#### 3.1.4 Dendritic cells

Dendritic cells (DCs) are the most common immune cells in the bladder after macrophages, comprising approximately 25% of all CD45^+^ cells (Bowyer et al., 2022). However, the role of DCs in UTIs is poorly understood. Multiple studies have demonstrated that DCs are dispensable for bacterial clearance during the innate immune phase of bladder infection. In CD11c-DTR mice depleted of all DC by injection of diphtheria toxin, the ability to clear UPEC from the bladder remains comparable to that in wild-type mice (Engel et al., 2006). This suggests that DCs may be primarily involved in coordinating immune responses rather than directly clearing pathogens.

Following ingestion of bacteria or bacterial antigens, adaptive immunity is initiated when dendritic cells mature and present antigens to T or B cells. However, mast cells-derived IL-10 during UPEC infection inhibits DCs activation but does not affect their migration to iliac lymph nodes. Immature DCs fail to induce significant antibody responses in lymph nodes (Bosteels and Janssens, 2025). Moreover, DCs and macrophages interact in phagocytosis of bacteria. In macrophage-depleted mice, DCs have enhanced phagocytosis of bacteria, suggesting that macrophages may preferentially sequester bacterial antigens, thereby reducing bacterial uptake by DCs and attenuating subsequent adaptive immune responses. This interplay may influence the magnitude and extent of both innate and adaptive immune responses (Mora-Bau et al., 2015).

#### 3.1.5 Natural killer (NK) cells

Natural killer (NK) cells are well-established contributors to innate immunity against tumors and viral infections, but their role in combating bacterial pathogens continues to emerge. Activated NK cells mediate target cell elimination by releasing cytotoxic mediators like perforin and granzymes, and secreting proinflammatory cytokines such as TNF-*α* and IFN-*γ* (O’Leary et al., 2006). Under homeostatic conditions, NK cells constitute less than 5% of bladder leukocytes (Yu et al., 2019). While their functional heterogeneity in the bladder remains poorly defined, murine UPEC infection models demonstrate NK cells expansion, implicating their importance in host defense.

During UPEC infection, stromal cell-derived factor 1 (SDF-1, also known as CXCL12), secreted by bladder epithelial cells, is a major recruiter of NK cells (Isaacson et al., 2017). A study has revealed that NK cells can be activated by the UPEC pilus protein FimH. However, NK cells apparently had no influence on the growth of either FimH^+^ or FimH^−^ UPEC bacterial strains, suggesting that their protective role is likely executed by orchestrating the broader immune response (Mian et al., 2010). It has also been demonstrated that NK cells can directly respond to UPEC and secrete TNF-α in the absence of HlyA, leading to decreased bacterial numbers *in vitro* and in the infected bladders. However, UPEC strains expressing HlyA can evade NK cells mediated immune clearance by disrupting the membrane integrity of NK cells, leading to their osmotic lysis or apoptosis (Gur et al., 2013). In contrast, a study has reported that depleting NK cells has no effect on bacterial clearance in UPEC infected bladder (Zychlinsky Scharff et al., 2019). More studies are required to delineate the role of NK cells in defending against UPEC infection in the bladder.

### 3.2 Adaptive immune cells

Although the extensive innate immune response to bladder infection is robust, the adaptive immune response is often limited. Therefore the recurrence rate is relatively high especially in adult women (Pigrau and Escolà-Vergé, 2020), suggesting impaired adaptive immunity in this population. While significant advances have been made in understanding the extensive innate immune responses to UTIs within the bladder, knowledge of the host and bacterial determinants influencing protective adaptive immunity remains limited.

#### 3.2.1 T cells

The bladder harbors diverse T cell subsets that play distinct roles in both innate and adaptive immunity during UTIs. Innate-like T cells, including *γ*δ T cells, natural killer T (NKT) cells, and mucosal-associated invariant T (MAIT) cells, represent unconventional T cell populations (Gao and Williams, 2015). However, the role of innate-like T cells is almost completely unknown in the context of UTIs. Adaptive immunity is mediated by CD8^+^ cytotoxic T cells and CD4^+^ helper T cells. In fact, the role of innate-like T cells is almost completely unknown in the context of UTIs.

Upon activation by antigen–MHC class II complexes, CD4^+^ T cells differentiate into distinct effector subsets—including Th1, Th2, and Th17 cells—each characterized by the secretion of a unique cytokine profile that mediates specialized functions within inflammatory responses (Wu and Abraham, 2021). Th1 and Th2 cells exhibit antagonistic functions in UTIs, with their balance determining bacterial clearance versus tissue regeneration. Th1 cells, characterized by secretion of IFN-γ, are essential for effective bacterial clearance by activating phagocytic functions of macrophages and CD8^+^ T cells and promoting neutrophil-mediated pathogen elimination (Wu et al., 2021). In contrast, Th2 cells, which produce cytokines such as IL-4, IL-5, and IL-13, drive tissue repair and epithelial regeneration but concurrently suppress Th1-mediated antimicrobial immunity (Lukens and Anand, 2020). In the bladder, this immune polarization is profoundly biased toward a Th2-dominated response, orchestrated by a unique subset of CD11c^+^CD301b^+^DCs that constitutively express OX40L. Upon UPEC infection, these DCs migrate to the draining lymph nodes, where they orchestrate the activation and polarization of naïve T cells toward Th2 phenotype (Wu et al., 2020). Consequently, recurrent infections entrench a Th2-biased immune profile that prioritizes rapid urothelial repair via growth factor production but compromises bacterial clearance, thereby favoring bacterial persistence (Lukens and Anand, 2020). Studies in *IL-4^−^/^−^* mice demonstrate that ablation of Th2 signaling enhances Th1 responses and reduces bacterial burdens, underscoring the antagonistic relationship between these subsets (Wu et al., 2020). This Th2-dominant microenvironment may represent an adaptive compromise to protect the bladder from urinary toxins; however, it predisposes to recurrent UTIs by limiting sterilizing immunity and permitting establishment of quiescent intracellular reservoirs.

Th17 and γδ T cells produce IL-17, a cytokine critical for neutrophil recruitment and biofilm disruption (Wu and Abraham, 2021). In the healthy mouse bladder, γδ T cells are a major source of IL-17, which may maintain the homeostasis of the bladder. After UPEC infection, bladder macrophages secrete CCL7 to recruit Th17 and γδ T cells, and IL-17 levels are significantly increased (Riding et al., 2022). The absence of IL-17A exacerbates bacterial burdens in the bladder and kidneys during UPEC infection. This deficiency is exploited by flagellated strains like CFT073, which leverage the compromised IL-17-mediated immunity to establish chronic bacterial reservoirs (Chamoun et al., 2020). The role of IL-17 in UTIs remains incompletely understood but may involve neutrophil recruitment. Post-UTIs analyses demonstrate elevated levels of neutrophil-recruiting chemokines in the bladder microenvironment (Riding et al., 2022), while IL-17-deficient mice exhibit significantly reduced neutrophil influx into the bladder (Sivick et al., 2010). These findings collectively suggest IL-17-mediated regulation of neutrophil trafficking during early UTIs pathogenesis. However, the mechanisms underlying γδ T cells activation via bacterial recognition or stress-induced signals during UPEC infection remain unclear.

#### 3.2.2 B cells

B cells in the bladder have received little attention due to lack of studies on adaptive immunity in UTIs. Plasma cells located in the healthy urinary tract lamina propria produce secretory IgA (sIgA), which contributes to immune defense and homeostasis, similar to its role in other mucosal surfaces (Corthesy, 2013). However, the specific mechanisms of how sIgA shapes the urinary microbiome and modulates immune responses in this environment are still being actively investigated.

A recent study describes a population of tissue-resident B cells in the kidney and bladder that play a role in tissue immune homeostasis and early infection response. The tissue resident B cells can promote macrophage anti-inflammatory polarization, consequently impairing bacterial clearance during UTIs. In contrast, B cell-deficient (μMT^−^) mice exhibited enhanced bacterial clearance, increased recruitment of neutrophils and monocytes, and pro-inflammatory polarization of macrophages. It suggests that a potential regulatory role of tissue resident B cells in early phase of bacterial infection (Suchanek et al., 2023). In a UPEC-induced cystitis model, bacterial loads are significantly lower in wild-type mice compared to Rag1^−^/^−^ counterparts, suggesting adaptive immunity in UTIs control. Moreover, albeit failed to be boosted with a second UTI, transient and modest germinal center (GC) B cells reactions establish by 4 weeks post-infection in bladder-draining lymph nodes (Hawas et al., 2023). Persistent bacteriuria and rUTIs are associated with chronic B cell infiltration in the bladder submucosa. These infiltrates also correlate with urothelial damage, including loss of umbrella cells and uroplakin expression, suggesting that B cells may contribute to both immune defense and tissue impairment (Schlager et al., 2011). However, the evidence for the role of B cells and humoral immunity in preventing or alleviating cystitis remains inconclusive.

Tertiary lymphoid structures (TLS) are ectopic lymphoid aggregates that form in non-lymphoid tissues under conditions of chronic inflammation or persistent antigen exposure (Bao et al., 2024). In the bladder mucosa of postmenopausal women with rUTIs, bladder TLS (bTLS) manifest as cystitis cystica lesions and contain well-organized B- and T-cell compartments with GCs, consistent with active cognate B–T cell interactions (Ligon et al., 2023b). Immunofluorescence analyses of patient biopsies demonstrate CD20 + B-cell follicles adjacent to CD3 + T cells, supported by CD21+ follicular dendritic cell networks, closely paralleling the structured bTLS observed in aged female mice (Ligon et al., 2020; Ligon et al., 2023b). Within these GCs, T follicular helper cells provide essential signals to drive B-cell activation, affinity maturation, and plasma cell differentiation—processes directly evidenced in murine bTLS, where they result in elevated urinary IgA (Ligon et al., 2020). In the postmenopausal setting of estrogen deficiency and TNF*α*-driven inflammaging, this B–T cell collaboration within bTLS may sustain chronic mucosal inflammation while impairing effective pathogen clearance (Ligon et al., 2023a). These findings position bTLS as a key site of dysregulated adaptive immunity in postmenopausal rUTIs and underscore the value of murine bTLS models for mechanistic insight and therapeutic development.

## 4 Trained immunity

For a long time, immunological memory was considered unique to the adaptive immune response. However, it is now established that pathogen exposure induces epigenetic modifications in host cells, altering their response to subsequent challenges (Netea et al., 2020). This concept is termed trained immunity. Trained immunity rests on two main pillars: epigenetic and metabolic reprogramming of cells. It operates by modifying chromatin accessibility, recruiting transcription factors, and ultimately altering the expression of genes regulating inflammatory responses (Fanucchi et al., 2021). These changes can either enhance host defense or facilitate bacterial persistence during subsequent infections. Recent research on UTIs has proposed the concept of epithelial-intrinsic trained immunity, providing crucial insights into UPEC-host interactions and the high recurrence rate of UTIs (Zhang et al., 2016; Aghagoli et al., 2023).

The outcome of an initial UTIs strongly predicts future recurrence risk, a finding substantiated by both animal models and clinical observations (O’Brien et al., 2016; Monack et al., 2015). In murine models, individuals with spontaneously resolved primary infections (resolved mice) exhibit resistance to rUTIs, whereas those developing chronic cystitis (sensitized mice) demonstrate high susceptibility (O’Brien et al., 2016). Mechanistically, resolved mice exhibit a rapid and transient inflammatory response mediated by TNF-α and COX-2, which promotes pathogen clearance and mucosal healing. In contrast, sensitized mice sustain persistent overexpression of TNF-α and COX-2, driving excessive neutrophil infiltration, mucosal injury, and chronic inflammation (Hannan et al., 2014). At the epigenetic level, resolved and sensitized mice harbor divergent chromatin accessibility profiles, leading to differential expression of caspase-1, stem cell pluripotency factors, and COX-2-dependent inflammatory mediators (Russell et al., 2023). Additionally, UTIs exert regulatory effects on bladder epithelial morphology and differentiation status. Specifically, this manifests as hyperproliferation of basal cells coupled with impaired regeneration of terminally differentiated superficial umbrella cells. Following recurrent or severe infections, the bladder epithelial cells lose characteristic stratified architecture (basal-intermediate-superficial layers) and specialized epithelial cell barrier function. This aberrant morphology constitutes the pathological basis for chronic inflammation, failed tissue repair, and predisposition to recurrent infection (Guo et al., 2023).

## 5 Concluding remarks

The bladder employs a multifaceted defense system comprising physical barriers, antimicrobial proteins, urothelial cells, and immune cells to preserve tissue integrity and counteract pathogenic invasion. The fate of an infection, whether it is resolved, becomes chronic, or recurs, hinges on the efficacy of this coordinated response. An immediate, robust innate immune reaction is crucial for early pathogen control; yet, the parallel urgency to restore the epithelial barrier and protect against toxic urinary solutes can force a premature attenuation of this pro-inflammatory response. This paradox may explain the frequent failure to develop sterilizing adaptive immunity, thereby underpinning the high rates of UTI recurrence.

Central to navigating this dilemma is the dynamic crosstalk between urothelial and immune cells, which orchestrates pathogen clearance while preserving barrier resilience. This interplay allows the bladder to maintain a delicate equilibrium: amplifying inflammatory responses to eliminate threats while initiating reparative program to restore homeostasis. Critically, the emerging concept of trained immunity provides a mechanistic framework for recurrence, demonstrating that prior infections can epigenetically reprogram bladder epithelial cells to mount either enhanced defensive or maladaptive inflammatory responses.

This insight underscores the need for therapeutic strategies that co-target bacterial virulence and host immune signaling to break the cycle of recurrence. Future studies must therefore prioritize the elucidation of specific epithelial-immune signaling networks and the functions of understudied immune cell subsets. Furthermore, the influence of sex, age, and hormonal factors on these processes demands rigorous investigation. Such work will pave the way for novel immunomodulatory therapies designed to bolster protective immunity while preserving tissue homeostasis, ultimately reducing the burden of recurrent disease.
